# Protective roles of thrombomodulin in cisplatin-induced nephrotoxicity through the inhibition of oxidative and endoplasmic reticulum stress

**DOI:** 10.1038/s41598-024-64619-y

**Published:** 2024-06-18

**Authors:** Hiroki Yamamoto, Yuko Ishida, Siying Zhang, Miyu Osako, Mizuho Nosaka, Yumi Kuninaka, Akiko Ishigami, Yuya Iwahashi, Miki Aragane, Lennon Matsumoto, Akihiko Kimura, Toshikazu Kondo

**Affiliations:** 1 Department of Forensic Medicine, Wakayama Medical University, 811-1 Kimiidera, Wakayama, 641-0012 Japan; 2 Department of Urology, Wakayama Medical University, Wakayama, Japan

**Keywords:** Cisplatin, Acute kidney injury, Thrombomodulin, Tubular cells, Mice, Biochemistry, Cell biology, Immunology, Nephrology

## Abstract

Cisplatin is an effective chemotherapeutic agent widely used for the treatment of various solid tumors. However, cisplatin has an important limitation in its use; currently, there is no method to ameliorate cisplatin-induced acute kidney injury (AKI). Thrombomodulin (TM) is well known not only for its role as a cofactor in the clinically important natural anticoagulation pathway but also for its anti-inflammatory properties. Here, we investigated the effects of TM in cisplatin-induced AKI. In mice intraperitoneally injected with 15 mg/kg cisplatin, TM (10 mg/kg) or PBS was administered intravenously at 24 h after cisplatin injection. TM significantly attenuated cisplatin-induced nephrotoxicity with the suppressed elevation of blood urea nitrogen and serum creatinine, and reduced histological damages. Actually, TM treatment significantly alleviated oxidative stress-induced apoptosis by reducing reactive oxygen species (ROS) levels in cisplatin-treated renal proximal tubular epithelial cells (RPTECs) in vitro. Furthermore, TM clarified cisplatin-induced apoptosis by reducing caspase-3 levels. In addition, TM attenuated the endoplasmic reticulum (ER) stress signaling pathway in both renal tissues and RPTECs to protect the kidneys from cisplatin-induced AKI. These findings suggest that TM is a potential protectant against cisplatin-induced nephrotoxicity through suppressing ROS generation and ER stress in response to cisplatin.

## Introduction

Acute kidney injury (AKI), which is found in 7–18% of hospitalized patients, is a common clinical syndrome characterized by renal dysfunction, resulting in the accumulation of metabolic wastes^1–3^. Cisplatin known as one of the renal toxins is the first-line chemotherapy prescribed to 10–20% of patients with cancer^4^. Several lines of accumulating evidence implied that cisplatin exerts nephrotoxicity^4^, ototoxicity^5^, neurotoxicity^5^, hepatotoxicity^6^, and gastrointestinal toxicity^7^ as harmful side effects. Actually, AKI occurred in 30% of patients treated with a single dose of cisplatin, eventually resulting in the discontinuation of administration^4^.

Recent studies have suggested that multiple signaling pathways and factors contribute to the development of cisplatin-induced tubular cell damage^8^. Mitochondria are a target organelle in cisplatin-induced kidney injury^9^. Cisplatin, accumulating in the mitochondria of renal proximal tubular cells, induces excessive production of reactive oxygen species (ROS), eventually leading to cisplatin-induced renal injury^10,11^. Therefore, a reduction in ROS levels can be effective in curing and relieving cisplatin-induced kidney damage.

Apoptosis of tubular epithelial cells exerts AKI through extrinsic and intrinsic apoptotic pathways and endoplasmic reticulum (ER) stress^12,13^. Mitochondrial dysfunction causes energy depletion and excessive production of ROS, leading to cell stress/injury and eventual ER stress-dependent apoptosis by activating the transcription of proapoptotic genes such as C/EBP homologous protein (CHOP)^12,14^. Thus, AKI pathogenically attribute to mitochondrial dysfunction and ER stress^14–16^.

Thrombomodulin (TM) is a single transmembrane, multidomain glycoprotein receptor for thrombin^17^ and provides a definitive molecular bridge that links inflammation and coagulation. TM also has well-defined anti-inflammatory functions^18^. Actually, TM administration attenuated cisplatin-induced intestinal toxicity though the suppression of cytokine production in intestinal epithelial cells of mice^7^, and Cheng and colleagues demonstrated that TM could regulate keratinocyte differentiation, eventually promoting wound healing^19^. Clinically, several lines of accumulating evidence implied that TM treatment showed therapeutic effects for acute pancreatitis^20^ and fulminant hepatitis^21^.

TM is predominantly expressed by vascular endothelial cells, and several other cell types, including neutrophils, synovial lining cells, and monocytes, are also known to express TM^22–24^. Clinically, recombinant human soluble TM (rTM) has been found effective for the treatment of disseminated intravascular coagulation^25^. Additionally the soluble form of TM attenuated ischemic renal injury and thromboembolism in rodent animals^26,27^.

In this study, we aimed to elucidate the effects and mechanisms of TM action on cisplatin-induced kidney injury in mice. Actually, we examined the biological effects and underlying mechanisms of TM in cisplatin-induced nephrotoxicity using both mice and primary tubular cells. Subsequently, we found protective effects of TM against AKI through the suppression of ROS generation and ER stress.

## Results

### TM-treated mice were resistant to cisplatin-induced AKI

**Figure 1:**
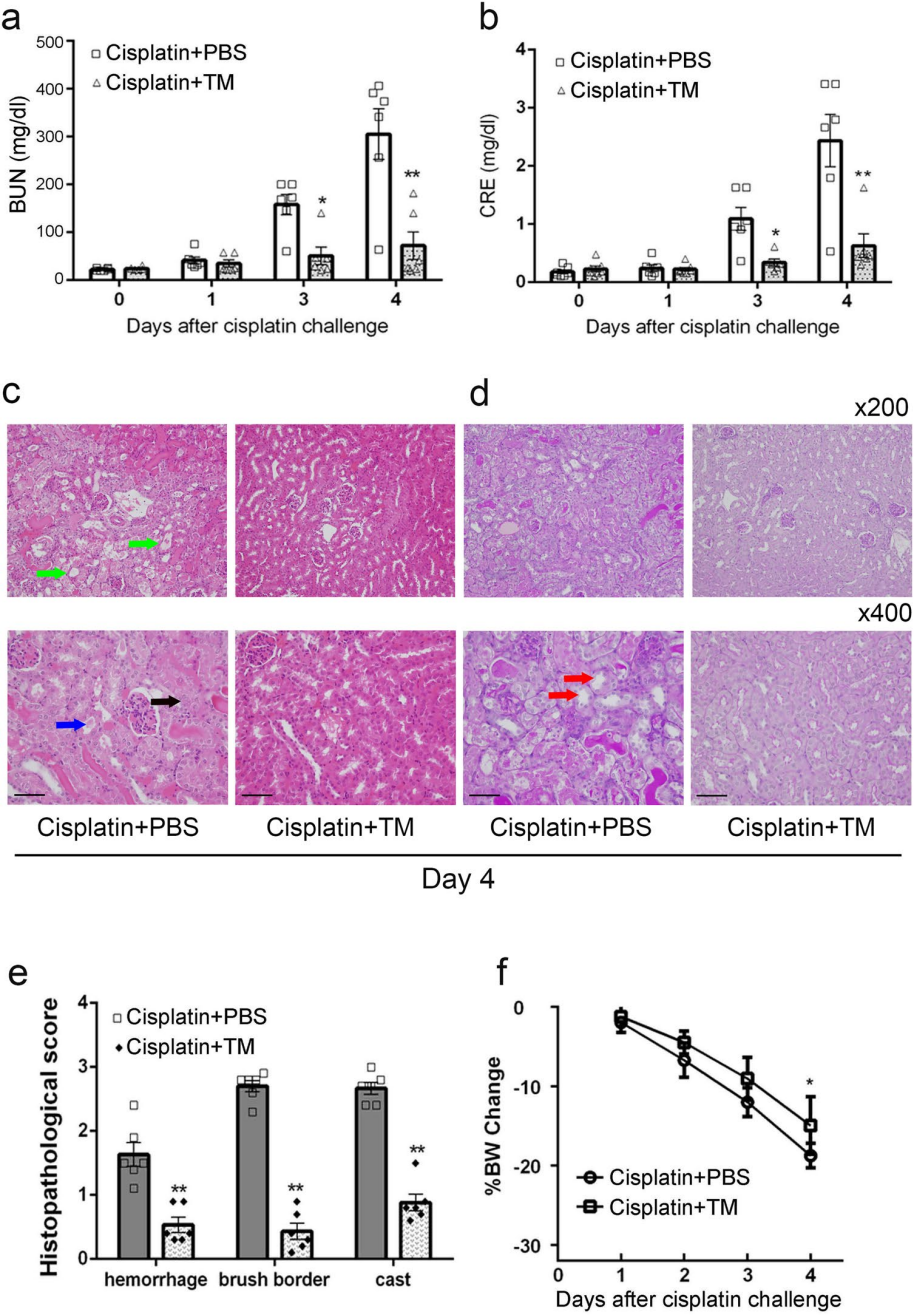
Thrombomodulin improved the cisplatin-induced AKI in mice. BUN (**a**) and CRE (**b**) levels of PBS-treated control (n = 6) and TM-treated mice (n = 6) one day after administration of 15 mg/kg cisplatin. All values represent means ± SEM. *P < 0.05; **P < 0.01, vs. PBS-treated control mice. Histopathological examinations (**c** HE; **d** PAS staining) on the kidneys from PBS-treated control and TM-treated mice at day 4 after cisplatin injection. The black arrow means necrosis; green arrow indicates tubular dilatation; blue arrow shows cast formation; red arrow points the disappearance of PAS-positive brush borders. Representative results from six individual animals are shown. Scale bars, 50 μm. (**e**) The histopathological score of the kidneys at day 4 after cisplatin injection was determined as described in “Materials and methods”. All values represent means ± SEM (n = 6). **P < 0.01, vs. PBS-treated control mice. (**f**) Percent body weight (BW) change from baseline was monitored each day after cisplatin injection. All values represent means ± SEM (n = 6). **P < 0.01, vs. PBS-treated control mice.

We examined the effects of TM treatment on cisplatin-induced nephrotoxicity. Cisplatin-administered mice were treated with PBS (control group) or TM. When mice administered cisplatin were treated with PBS, serum levels of BUN and CRE were significantly elevated at day 3 and 4 after cisplatin challenge. On the contrary, therapeutic TM treatment significantly suppressed the elevation of serum BUN and CRE levels in cisplatin-challenged mice (Fig. 1a,b). In control group, mice exhibited severe histopathological alterations such as tubular dilatation, cast formation, and epithelial desquamation with interstitial hemorrhages (Fig. 1c), as well as the disappearance of PAS-positive brush borders in damaged tubules (Fig. 1d). On the contrary these histopathological alterations were significantly attenuated in the TM-treated mice (Fig. 1c–e). In addition, the rate of weight loss was significantly lower in the TM group than that in the control group (Fig. 1f). These observations implied that TM treatment would alleviate cisplatin-induced nephrotoxicity.

### TM was not essentially involved in leukocyte recruitment during cisplatin-induced AKI

**Figure 2:**
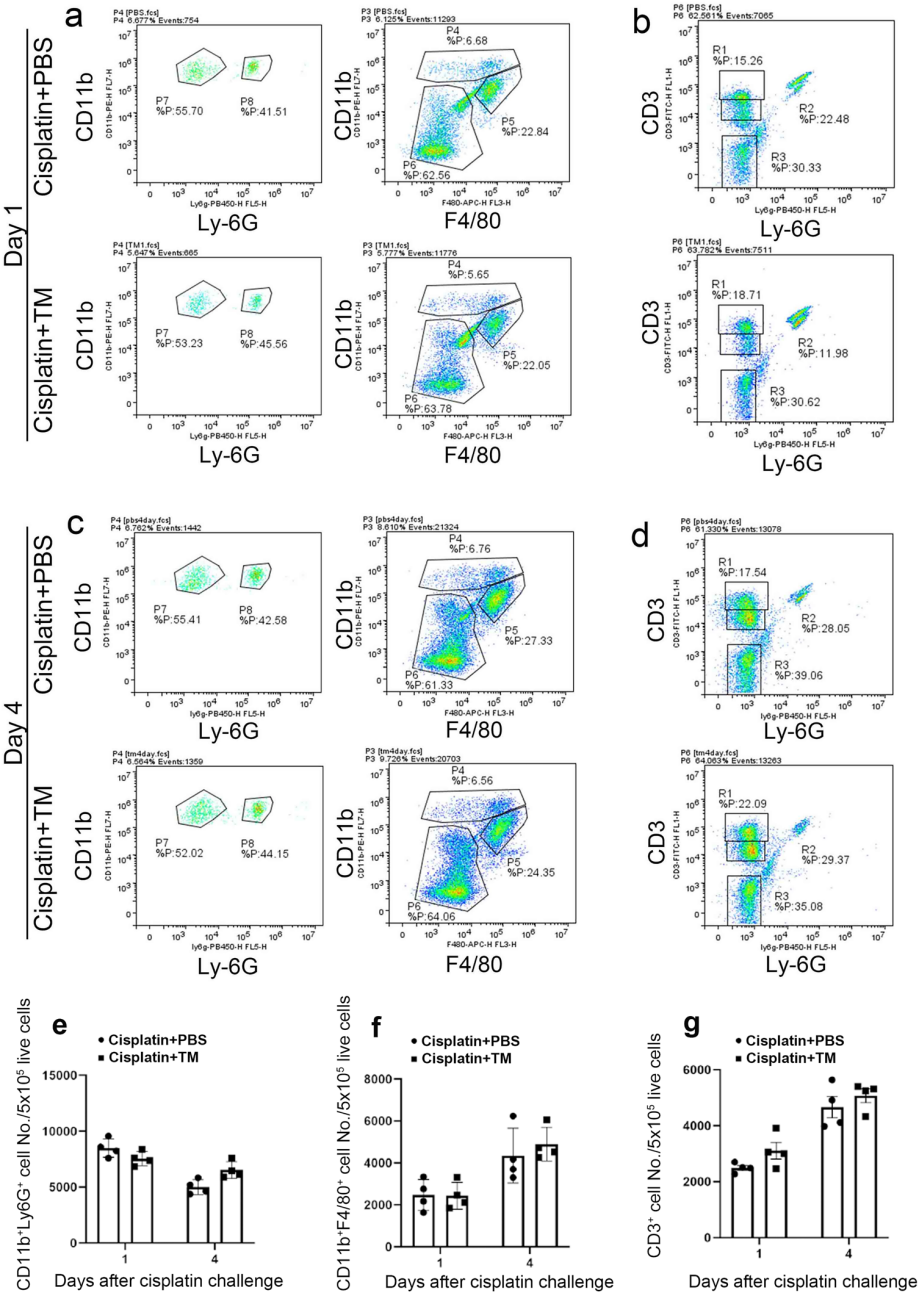
Evaluation of leukocyte recruitment after TM or PBS treatment during cisplatin-induced AKI. Flow cytometric analysis of CD11b^+^Ly6G^+^ neutrophils (P8), CD11b^+^F4/80^+^ macrophages (P5) (**a**,**c**), and CD3^+^ lymphocytes (R1) (**b**,**d**) in kidney tissue 1 (**a**,**b**) and 4 (**c**,**d**) days after cisplatin administration. Representative results from six independent experiments are shown here. The cell number of CD11b^+^Ly6G^+^ neutrophils (**e**), CD11b^+^F4/80^+^ macrophages (**f**), and CD3^+^ lymphocytes (**g**) after TM or PBS treatment during cisplatin-induced AKI.

Several lines accumulating evidence implied that the accumulation of leukocytes such as neutrophils, macrophages and T cells was one of the injurious factors in AKI^28–33^. Thus, we examined that the effects of TM on intrarenal leukocyte recruitment after cisplatin challenge. We determined leukocyte subsets as CD11b^+^Ly-6G^+^ neutrophils, CD11b^+^F4/80^+^ macrophages and CD3^+^ T cells by FACS analyses. In both control and TM groups, cisplatin treatment enhanced intrarenal recruitment of CD11b^+^Ly-6G^+^ neutrophils, CD11b^+^F4/80^+^ macrophages and CD3^+^ T cells at day 2 and 5 (Fig. 2). However, there were no significant differences in the intrarenal recruitment of each leukocyte subset between control and TM groups (Fig. 2). These observations implied that TM had no effects on leukocyte recruitment in cisplatin-induced AKI.

### TM up-regulated the expression of kidney protective molecules, IFN-γ and HO-1, in cisplatin-induced AKI

**Figure 3:**
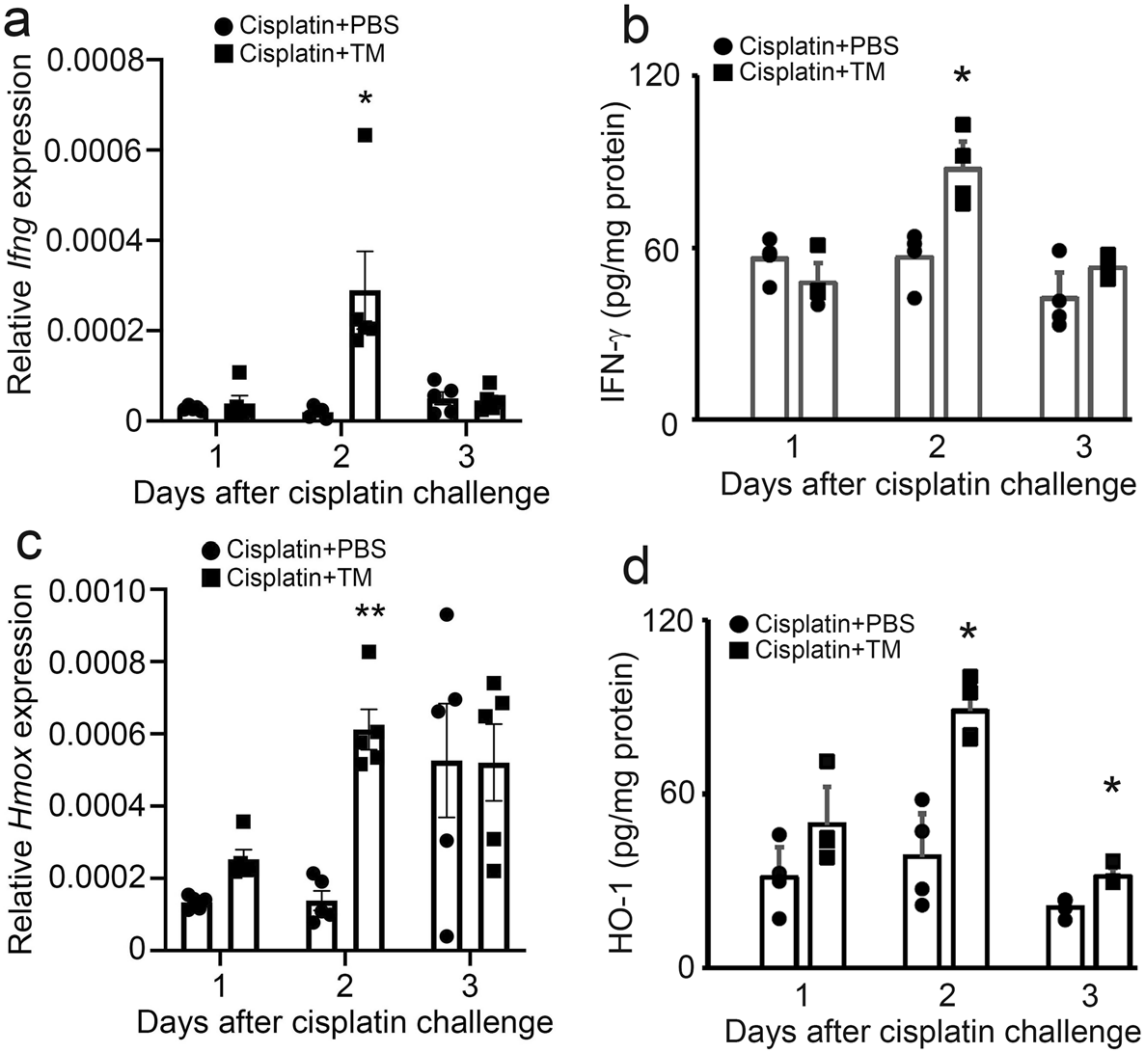
The effects off TM treatment on intrarenal gene and protein expression of IFN-γ (**a**,**b**) and HO-1 (**c**,**d**) during cisplatin-induced AKI. The gene expression of *Ifng* (**a**) and *Hmox* (**c**) was determined by real-time RT-PCR. All values represent means ± SEM (n = 5). *P < 0.05, **P < 0.01 versus PBS-treated control mice at the same time points. The protein contents of IFN-γ (**b**) and HO-1 (**d**) were determined by ELISA. Each value represents mean ± SEM (n = 4). *P < 0.05 versus PBS-treated control mice at the same time points.

Our previous study showed that IFN-γ played a protective role in cisplatin-induced AKI by increasing the viability of renal tubular cells^34^. In addition, Burks et al. demonstrated that IFN-γ stimulated the production of the anti-inflammatory cytokine IL-10 to ameliorate cisplatin-induced AKI^35^. In this study, we found that TM treatment more upregulated intrarenal IFN-γ expression at both gene and protein levels, compared with PBS treatment (Fig. 3a,b). Moreover, heme oxygenase (HO)-1 is a cytoprotective enzyme that catalyzes the breakdown of heme into biliverdin, carbon monoxide, and iron. The beneficial effects of HO-1 expression are not merely due to the degradation of the pro-oxidant heme but also due to the by-products that have potent protective effects, including antioxidant, anti-inflammatory, and pro-survival properties^36,37^. Bolisetty et al. demonstrated that although deletion of HO-1, specifically in the proximal tubules, aggravates structural and functional damage during cisplatin nephrotoxicity, selective overexpression of HO-1 in the proximal tubules plays a protective role^38^. Moreover, several lines of evidence indicate that upregulated HO-1 plays a protective role against cisplatin-induced AKI in mice^39,40^. Actually, the intrarenal HO-1 expression was more enhanced in TM-treated mice than in PBS-treated ones on day 2 (Fig. 3c,d). Thus, TM treatment may play a protective role in the development of cisplatin-induced AKI by upregulating the expression of renoprotective factors such as IFN-γ and HO-1 expression.

### TM treatment suppressed the expression of proinflammatory cytokines

**Figure 4:**
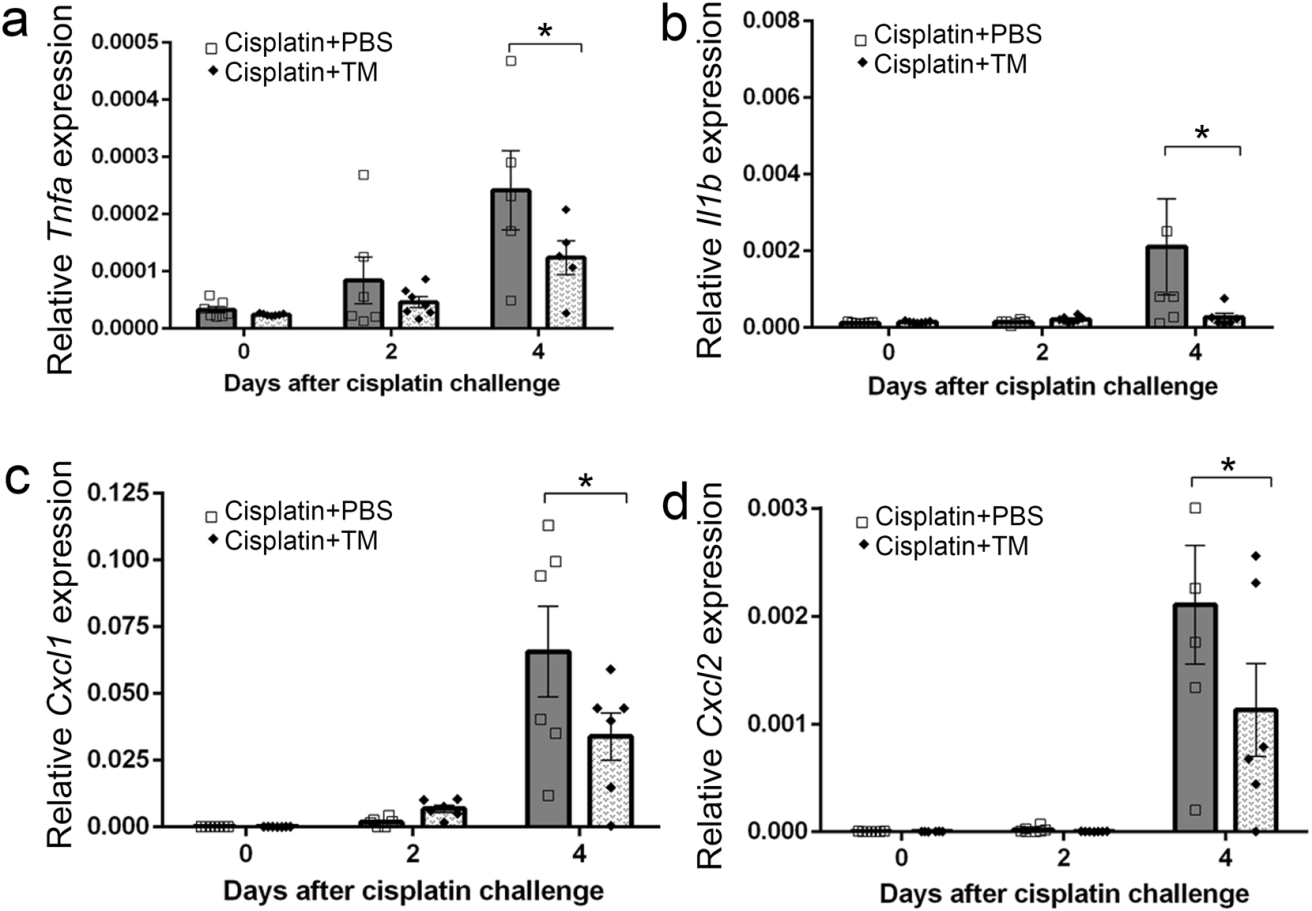
The gene expression of inflammatory cytokines and chemokines after TM or PBS treatment during cisplatin-induced AKI (**a** *Tnfa*, **b** *Il1b*, **c** *Cxcl1*, **d** *Cxcl2*). Real-time RT-PCR was performed on total RNA extracted from the kidney at indicated time intervals. All values represent means ± SEM (n = 6). **P < 0.01, vs. PBS-treated control mice.

Inflammatory responses play a major role in the pathophysiology of cisplatin-induced AKI^41^. In vivo studies have shown that an increase in the intrarenal levels of proinflammatory cytokines is associated with cisplatin-induced AKI^42–46^. In the present study, cisplatin administration enhanced the intrarenal expression of *Tnfa*, *Il1b*, *Cxcl1* and *Cxcl2* whereas TM treatment significantly suppressed those expression on day 4 (Fig. 4). Thus, these observations implied that TM would have a protective role in cisplatin-induced AKI through the suppression of inflammatory responses.

### TM limited intracellular ROS increase induced by cisplatin

**Figure 5:**
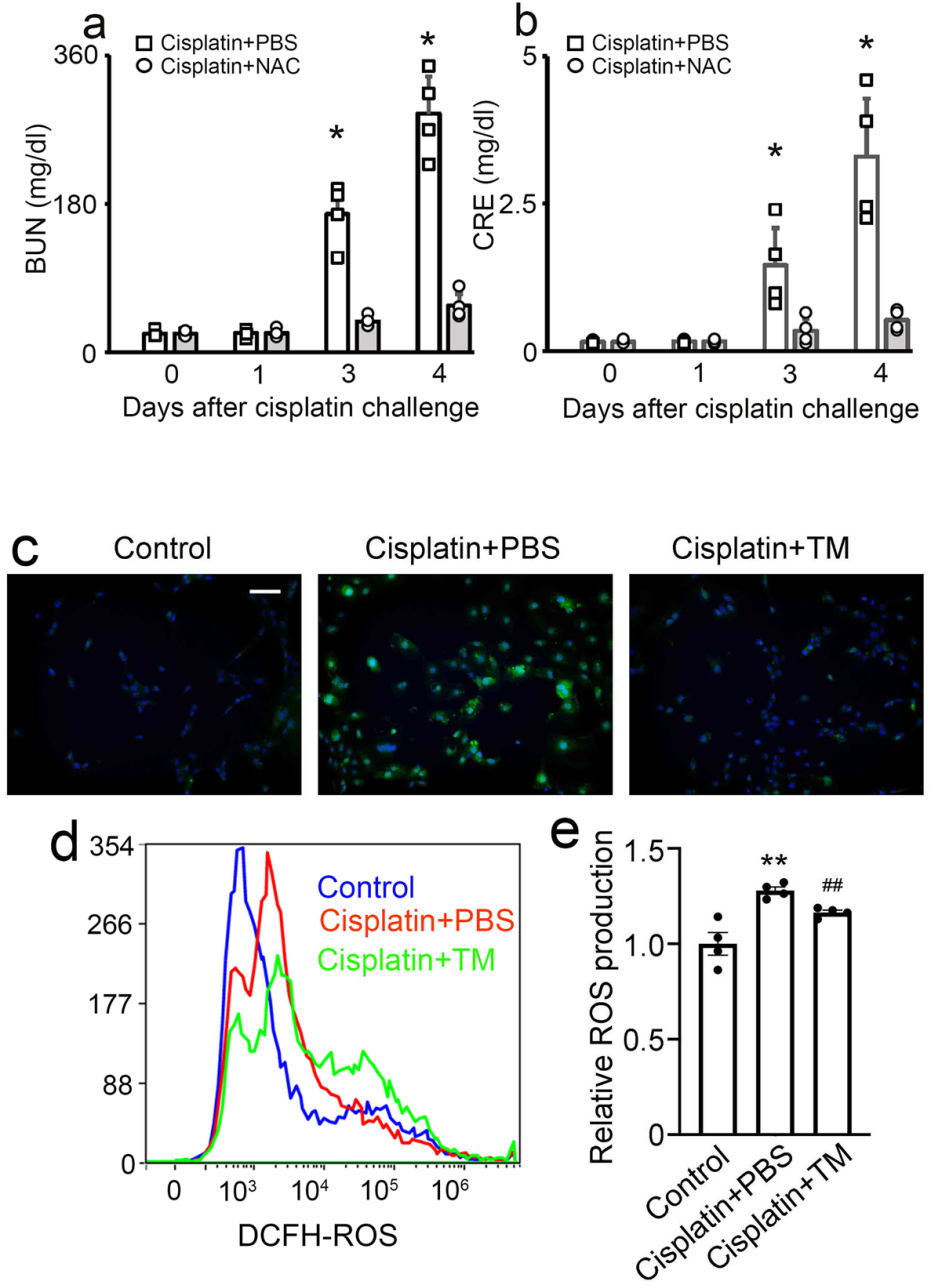
The effects of *N*-acetylcysteine (NAC) on cisplatin-induced kidney injury. Serum BUN (**a**) and CRE (**b**) levels were evaluated at the indicated time intervals. All values represent means ± SEM (n = 4). *P < 0.05 vs. PBS-treated control mice. The effects of TM on ROS production in RPTECs treated with cisplatin. (**c**) Cellular ROS level was visualized by fluorescence microscopy, and representative results from five independent experiments are shown here (scale bar, 20 μm). (**d**) Cells were incubated with ROS probe at 4 h after treatment and subjected to flow cytometry analysis. Representative results from five independent experiments are shown here. (**e**) Relative ROS production from five independent experiments. All values represent means ± SEM. (**P < 0.01 vs. Control; ^##^*P* < 0.01 vs. PBS group).

Cisplatin caused the excessive production of reactive oxygen species (ROS) in tubular cells of the kidney, eventually resulting in renal damage^10,11^. Therefore, a reduction in ROS levels can be effective in curing and relieving cisplatin-induced kidney damage. In line with this, the administration of *N*-acetylcysteine (a ROS inhibitor) reduced cisplatin-induced renal injury as evidenced by serum levels of BUN and CRE (Fig. 5a,b). In the next series, we examined the effects of TM on intracellular ROS levels using renal proximal tubular epithelial cells (RPTECs). Intracellular ROS levels in cisplatin-treated RPTECs were measured using DCFH-DA fluorescent probes. When RPTECs were exposed to cisplatin, intracellular ROS levels were significantly increased. However, the supplementation with TM significantly suppressed intracellular ROS production in cisplatin-treated RPTECs (Fig. 5c). In line with this, FACS analyses revealed that TM treatment reduced the ROS levels in cisplatin-treated RPTECs (Fig. 5d,e). Collectively, these observations implied that TM administration inhibited ROS production, eventually resulting in the reduction of cisplatin-induced nephrotoxicity.

### TM inhibited cisplatin-induced ER stress in mice

**Figure 6:**
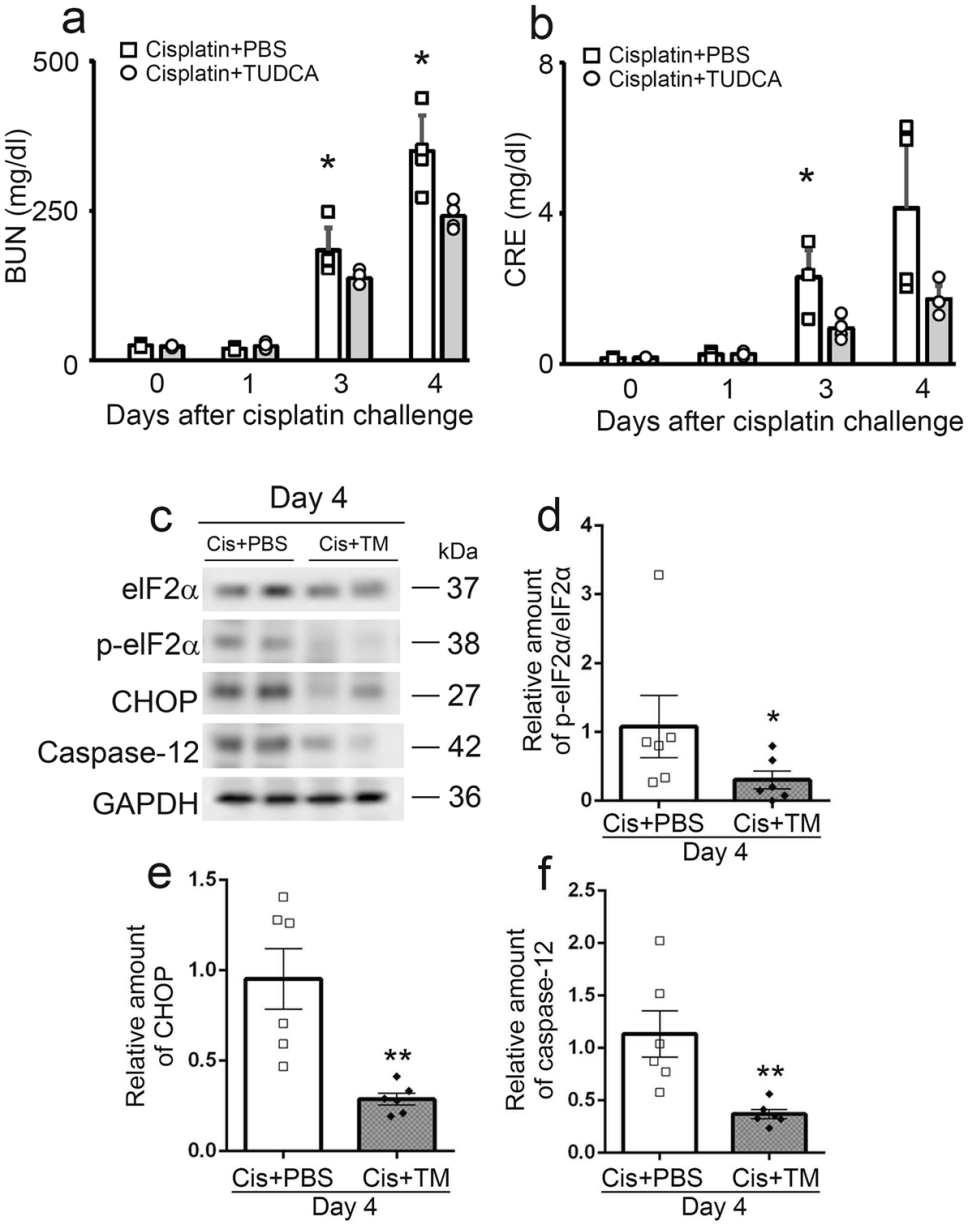
The effects of tauroursodeoxycholic acid (TUDCA) on cisplatin-induced kidney injury. Serum BUN (**a**) and CRE (**b**) levels were evaluated at the indicated time intervals. All values represent means ± SEM (n = 4). *P < 0.05 vs. PBS-treated control mice. TM alleviated cisplatin-induced ER stress in kidney. (**c**) The expression of key ER stress proteins such as eIF2α, CHOP, and caspase-12 was analyzed by western blotting with specific primary antibodies. GAPDH levels serve as load control. The original blots are presented in Supplementary Fig. S1. Expression levels of p-eIF2α/eIF2α (**d**), CHOP (**e**), and caspase-12 (**f**) measured by immunoblot analysis. All values represent means ± SEM (n = 6). *P < 0.05; **P < 0.01, vs. PBS-treated control mice.

Cisplatin can induce ER stress, eventually resulting in renal cell damages such as apoptosis^47^. Thus, we examined the effects of tauroursodeoxycholic acid (TUDCA), known as an ER stress inhibitor, on cisplatin-induced nephrotoxicity in mice. In line with the previous report^47^, mice treated with TUDCA showed less increases in BUN and CRE after cisplatin challenge (Fig. 6a,b). Mechanistically, ER stress activates the caspase-12-dependent CHOP apoptotic pathway. The purpose of detecting ER stress-related proteins in kidney tissues was to evaluate the regulatory effects of TM on ER stress. Actually, the expression levels of eIF2α, CHOP, and caspase-12 significantly increased in the kidneys of the PBS group. Compared with the PBS group, TM treatment significantly reduced the expression levels of eIF2α, CHOP, and caspase-12 in the kidney after cisplatin administration (Fig. 6c–f). These observations indicated that TM exerted a protective effect against cisplatin-induced ER stress.

### TM reduced cisplatin-induced apoptosis in the kidney

**Figure 7:**
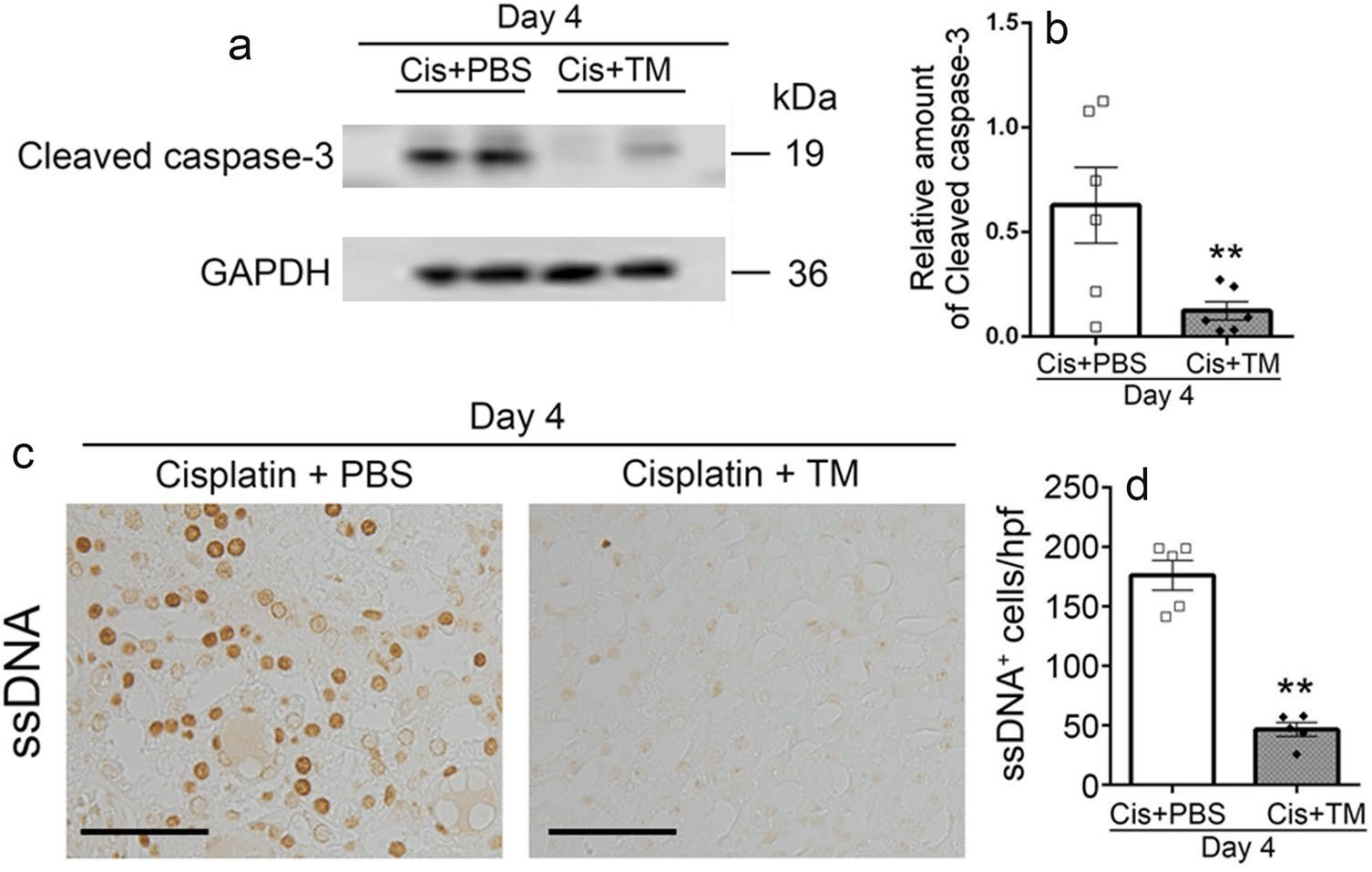
TM reduced cisplatin-induced apoptosis in mice. (**a**) The protein expression of cleaved caspase-3 was analyzed by western blotting with specific primary antibodies. GAPDH levels serve as load control. The original blots are presented in Supplementary Fig. S2. (**b**) Relative amount density of cleaved caspase-3. All values represent means ± SEM (n = 6). *P < 0.05; **P < 0.01, vs. PBS-treated control mice. (**c**) Kidney tissue was stained with anti-ssDNA. Scale bar, 20 μm. (**d**) The number of ssDNA^+^ apoptotic cells were determined. All values represent means ± SEM (n = 5). *P < 0.05; **P < 0.01, vs. PBS-treated control mice.

Several lines of accumulating evidence demonstrated that ER stress-induced activation of caspase-12 cleaved procaspase-9 to activate procaspase-3, resulting in the promotion of apoptosis^28,29^. Manohar and Leung indicated that tubular cell apoptosis is responsible for cisplatin-induced kidney injury^30^. Thus, we examined the effects of TM on renal cell apoptosis after cisplatin challenge. Actually, the intrarenal level of cleaved caspase-3 was significantly reduced in TM treatment group, compared with PBS treatment one (Fig. 7a,b), indicating that TM might effectively prevent cisplatin-induced apoptosis of renal cells. Expectedly, at 4 days after cisplatin administration, a large number of ssDNA^+^ apoptotic cells were observed in PBS-treated mice, whereas the number of apoptotic cells was significantly reduced in TM-treated mice (Fig. 7c,d). These observations indicated that TM rendered renal cells highly resistant to cisplatin-induced apoptotic signals.

### Effect of TM on cisplatin-induced apoptosis and ER stress in RPTECs

**Figure 8:**
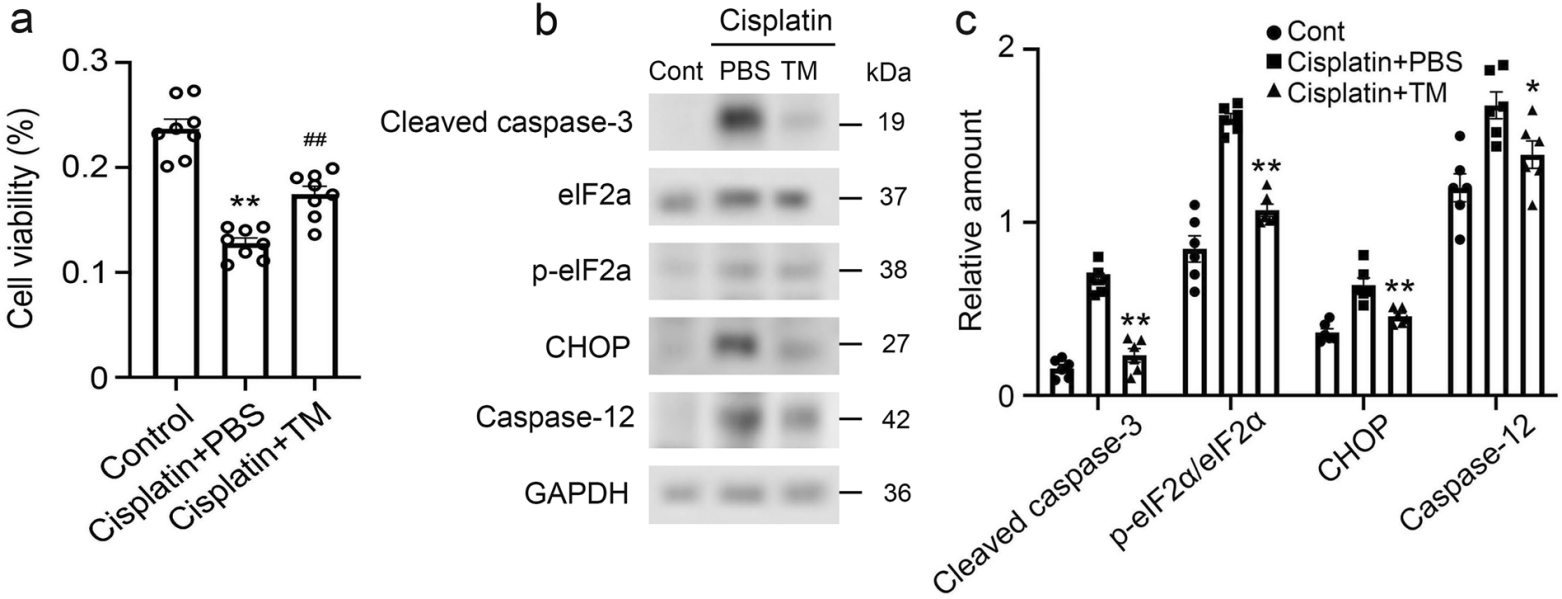
Western blotting analysis of eIF2α, CHOP, caspase-12, and cleaved caspase-3 in renal proximal tubular epithelial cells (RPTECs) treated with cisplatin and PBS or TM. (**a**) The cell viability of RPTECs was assessed at 12 h after the treatment with cisplatin (50 μmol/ml) and PBS or TM (1 μg/ml). All values represent means ± SEM (n = 8 experiments). **P < 0.01 vs. Control; **P < 0.01 vs. PBS group. (**b**) The expression of eIF2α, p-eIF2α, CHOP, and caspase-12 was analyzed by western blotting with specific primary antibodies. GAPDH levels serve as load control. Representative results from six independent experiments are shown. The original blots are presented in Supplementary Fig. S3. (**c**) Expression levels of cleaved caspase-3, p-eIF2α/eIF2α, CHOP, and Caspase-12 measured by immunoblot analysis. All values represent means ± SEM (n = 6). *P < 0.05; **P < 0.01, vs. PBS-treated control mice.

Our in vivo observations implied that TM would alleviate cisplatin-induced ER stress and eventual tubular cell apoptosis, suppressing cisplatin nephrotoxicity. Thus, we examined the effect of TM on primary RPTECs treated with cisplatin. The effects of TM on cisplatin-induced damage to RPTECs were assessed by adding TM or PBS to the culture medium. In line with in vivo observations, the addition of TM significantly increased cell viability in cisplatin-treated RPTECs, indicating that TM played a protective role against cisplatin-induced cytotoxicity (Fig. 8a). Furthermore, apoptotic signaling molecules, such as the protein level of cleaved caspase-3, were profoundly increased in PBS-treated RPTECs, whereas these pro-apoptotic signaling molecules were decreased in TM-treated RPTECs (Fig. 8b). In line with in vivo observations, in cisplatin-treated RPTECs, TM treatment markedly decreased the expression of cleaved caspase-3 as well as that of ER stress-related molecules such as phosphorylated eIF2a, CHOP and caspase-12 (Fig. 8c). These observations indicating that TM would suppress ER stress and eventually apoptosis, resulting in the alleviation of cisplatin nephrotoxicity.

## Discussion

Cisplatin is the drug of prime choice in platinum-based chemotherapy for the treatment of various cancers. Its exposure is frequently associated with nephrotoxicity caused by ROS-mediated ER stress, inflammation, and apoptosis^30,48^. Our results revealed that cisplatin administration led to a significant increase in cell death, ROS generation, ER stress induction, and inflammatory responses in mouse kidneys and RPTECs. These altered RPTECs toxicity parameters and mouse kidney injury were markedly alleviated by TM treatment, suggesting that TM exerted operative resistance against cisplatin-induced nephrotoxicity. To the best of our knowledge, this is the first study to demonstrate the potential protective effects and possible mechanism of action of TM against cisplatin-induced nephrotoxicity.

We found that serum BUN and CRE levels significantly increased after treatment with 15 mg/kg cisplatin. However, post-treatment with TM dramatically reduced serum BUN and CRE levels, resulting in recovery from kidney injury. Moreover, histopathological examination of mouse kidneys confirmed the protective effects of TM against cisplatin-induced renal damage. In addition, immunohistochemical analysis using anti-ssDNA suggested that TM significantly reduced cell apoptosis, indicating that TM protected the kidney via its anti-apoptotic effects. This study identified the beneficial effects of TM in preventing renal tubular damage and dysfunction. However, it remains unclear whether this anti-apoptotic effect was directly or indirectly mediated by TM.

The pathophysiology of cisplatin-induced AKI involves various molecular and cellular mechanisms, including increased oxidative stress, inflammation, and apoptosis^8,49^. Several studies have shown that antioxidants exert protective effects against cisplatin-induced nephrotoxicity through their effects on inflammation and oxidative stress^50^. Oxidative stress is a characteristic of cisplatin-induced nephrotoxicity and an important factor in the development and progression of other associated damage mechanisms, such as mitochondrial dysfunction, inflammation, and ER stress^30,51,52^. ROS are naturally generated and regulate cellular function^53^. However, excessive ROS can damage proteins, modify their function, and disrupt ER homeostasis^54,55^. ROS overproduction can be sensed by the ER, which is highly sensitive to changes in ROS levels through redox sensors, such as the thiol groups of cysteine residues^52,56^, causing misfolded protein accumulation and consequently increasing ROS production, which leads to chronic stress and induces apoptosis^52,53,55^. Because cisplatin mainly destroys renal tubular cells and induces renal failure^57^, we evaluated the viability and ROS generation of RPTECs to evaluate the effects of TM. The data showed that TM could improve the viability of cisplatin-treated RPTECs and reduce the production of ROS, which is consistent with the in vivo data and indicates that TM protects renal tubular cells.

Cisplatin-induced apoptosis of renal tubular cells is mainly associated with the mitochondria-mediated intrinsic pathway, the death receptor-mediated extrinsic pathway, and ER stress, all of which promote apoptosis through downstream caspase-3^58^. To determine whether ER stress-mediated apoptosis was involved, we analyzed the expression of caspase-3 in both kidney tissues and RPTECs using western blotting and found that TM inhibited the upregulation of cisplatin-induced caspase-3, confirming that TM could inhibit cisplatin-induced ER stress-mediated apoptosis.

ER stress is a novel apoptotic pathway that regulates the toxic effects of drugs on cells^59^. Recent studies have shown that cisplatin induces ER stress, upregulates multiple ER stress markers, activates caspase-3, and promotes cell damage and apoptosis^60^. ER pathway regulates cisplatin-induced apoptosis in renal tubular cells. Caspase-12 on the ER surface is a pivotal promoter of ER stress. Cisplatin activates caspase-12 in an epithelial cell line of the proximal tubule of the porcine kidney and induces apoptosis^61^. When numerous misfolded or unfolded proteins accumulate in the ER, PERK dissociates from GRP78/BIP and activates the unfolded protein-response signaling cascade. After dissociation, PERK and GRP78 autophosphorylate and oligomerize, activate eIF2a, reduce protein synthesis, and reduce the amount of protein entering the ER^62^. Subsequently, eIF2a phosphorylation can upregulate the expression of ATF4, which activates CHOP. During ER stress, CHOP expression is significantly upregulated, and it is mainly distributed in the nucleus^63^. As a transcription factor, CHOP is involved in regulating the expression of the downstream target gene Bcl-2, which affects cell growth and death. Under ER stress, the activated caspase-12 is transferred to the cytoplasm and the downstream signaling protein caspase-9 is shear-activated, which, in turn, activates the core protein caspase-3 and induces apoptosis, finally completing the caspase-12-mediated apoptotic response^64^. The protective effect of TM on renal cells involves reducing the apoptosis of renal cells and the amount of unfolded and misfolded proteins retained in the ER stress. To explore the effects of TM on ER stress in cisplatin-induced renal toxicity in mice, we examined the expression of ER stress-related and apoptotic proteins. The results showed that cisplatin promoted the expression of p-eIF2a, eIF2a, and CHOP, but these protein levels decreased significantly in the TM group compared with those in the PBS group. Thus, the protective effect of TM against cisplatin-induced AKI in mice was related to the inhibition of ER stress.

Oxidative stress is one of the most important factors involved in cisplatin-induced acute renal failure, followed by ROS accumulation^65,66^. Oxidative stress triggers the release of Nrf2 from the Nrf2–Keap1 complex, thereby inducing the expression of Nrf2 and its related downstream genes. Nrf2 is a transcription factor that upregulates the expression of genes encoding anti-oxidant-related proteins, including HO-1, which protects tissues by alleviating the oxidative damage caused by free radicals^67^. HO-1 is a redox-sensitive microsomal enzyme that catalyzes the degradation of heme to biliverdin, iron, and carbon monoxide^68^. HO-1 is activated in the kidneys following cisplatin treatment^69^. HO-1 deficient mice are markedly sensitive to cisplatin-induced AKI. In addition, HO-1 overexpression significantly ameliorates cisplatin-induced apoptosis^70^. Moreover, cells overexpressing HO-1 have significantly low levels of ROS and are protected from cisplatin cytotoxicity^71^. Our observations implied that TM could upregulate the gene expression of HO-1, eventually alleviating cisplatin-induced AKI via an antioxidant pathway.

Caspase-12 is primarily located on the cytoplasmic face of the ER and is highly expressed in renal proximal tubular epithelial cells^42^. Caspase-12 can be specifically activated via ER stress but not via membrane or mitochondrial signals. It was previously demonstrated that caspase-12-deficient mice do not respond to ER stress-induced apoptosis^72^. Therefore, caspase-12 is considered a marker of ER stress-induced apoptosis. In our study, ER stress and apoptosis were triggered in cisplatin-induced nephrotoxicity, as demonstrated by the significant increase in caspase-12 levels. In contrast, the TM group showed a significant reduction in caspase-12 levels. These results indicate that TM could attenuate ER stress-induced apoptosis.

Inflammation plays a critical role in the development of cisplatin-induced kidney injury^73^. Increased expression levels of inflammatory cytokines, such as TNF-α and IL-1β, have been reported in cisplatin-induced kidney injury in mice^42^. Overexpression of these inflammatory cytokines leads to kidney tissue damage^74^. Previous studies have shown that the inhibition of these cytokines attenuates cisplatin-induced kidney tissue injury^75^. In addition, treatment with anti-TNF-α or TNF-α inhibitors and TNF-α deficiency in mice ameliorate cisplatin-induced AKI, with blunted cisplatin-induced increase in IL-1β expression^42^. Therefore, these inflammatory cytokines and chemokines play detrimental roles in the pathogenesis of cisplatin-induced AKI, and TM treatment could suppress the expression of these molecules. In contrast, TM treatment could enhance the expression of renoprotective molecules, such as IFN-γ, in the kidney^34^ without no alteration in leukocyte infiltration after cisplatin administration. Taken together, these data suggest that TM inhibits DNA damage and apoptosis, indicating that it may be viable as a nephroprotectant.

This study reports the protective effects of TM against cisplatin-induced kidney damage. Both in vitro and in vivo data demonstrated that TM inhibited ER stress-mediated apoptosis to protect the kidney and renal tubular epithelial cells from injury and apoptosis via the removal of ROS. This study indicated the potential clinical application of TM in the prevention of cisplatin-induced kidney injury.

## Materials and methods

### Reagents and antibodies (Abs)

Cisplatin, *N*-acetylcysteine (NAC) and tauroursodeoxycholic acid (TUDCA) were purchased from Sigma-Aldrich (St. Louis, MI), Abcam (Waltham, MA) and Selleck Biotech (Kanagawa, Japan), respectively. Recombinant human soluble thrombomodulin (TM) was a gift from Asahi Kasei Pharma Corporation (Tokyo, Japan). The following monoclonal or polyclonal Abs were used for immunohistochemical or western blotting analysis: rabbit ssDNA pAbs (Immuno-Biological Laboratories, Gunma, Japan), rabbit anti-cleaved caspase-3 pAbs, rabbit phospho-eIF2α (Ser51) mAb, rabbit anti-eIF2α (D7D3) mAb, mouse anti-CHOP mAb, rabbit anti-caspase-12 mAb and rabbit anti-GAPDH mAb (Cell Signaling Technology, Danvers, MA). FACS were performed with the following Abs: eFluor 450-labeled rat anti-mouse Ly-6G (eBioscience, San Diego, CA), FITC labeled rat anti-mouse CD3e (eBioscience), APC labeled rat anti-F4/80 (Biolegend Japan, Tokyo, Japan) and Brilliant Violet 510-labeled rat anti-mouse CD45 (Biolegend, Japan).

### Experimental animals and acute kidney injury model

Eight- to 10-week-old male BALB/c mice were obtained from Sankyo Laboratories (Tokyo, Japan). All animals were maintained in a specific pathogen-free environment and subjected to regular light–dark cycle at temperature of 25 °C, and freely accessed to food and water in the animal house of Wakayama Medical University. In order to develop AKI model, mice were given a single intraperitoneal injection of cisplatin (15 mg/kg) on the first day. At 1 day after cisplatin treatment, tail intravenous injection of TM (10 mg/kg) or PBS (200 μl) were given to each mouse^76^. In another series, NAC (50 mg/kg) or TUDCA (250 mg/kg) was intraperitoneally administered mice twice a day at 0–3 days after cisplatin injection. Kidney tissues and blood samples were obtained at the indicated time intervals after cisplatin challenges.

### Measurement of serum BUN and CRE levels

At the indicated time intervals after cisplatin challenge, serum levels of BUN and CRE were determined with a Fuji DRI-CHEM 5500 V (Fuji Medical System, Tokyo, Japan), according to the manufacturer’s instructions.

### Histopathological analyses

Kidney tissues were obtained at 4 days after cisplatin challenge and were fixed in 4% formaldehyde buffered with PBS (pH 7.2), followed by making paraffin-embedded sections (4 μm thickness). The deparaffinized sections were subjected to HE staining or periodic acid-Schiff staining. For the semi-quantitative evaluation of renal damage, hemorrhages, the disappearance of periodic acid-Schiff-positive brush border, and cast formation were graded in 10 randomly chosen fields at a magnification of ×200. Subsequently, renal morphological changes were scored on a scale of 0–3: 0, normal; 1, slight; 2, moderate; and 3, severe^34^. The average of all of the regions was calculated in each sample. All measurements were recorded without a prior knowledge of the experimental procedures.

### Immunohistochemical staining

After the immersion of deparaffinized sections in 0.3% H_2_O_2_ in methanol for 30 min, the sections were further incubated with PBS containing 1% normal serum and 1% bovine serum albumin to reduce nonspecific reactions. The sections were incubated with anti-ssDNA pAbs at a concentration of 1 μg/ml at 4 °C overnight. After the incubation the sections with HRP-conjugated goat anti-rabbit IgG, immune complexes were visualized using Tyramide Amplification Buffer Plus (22029, Biotium, Fremont, CA) with Tyramide Signal Amplification Kits (33020, Biotin-XX, Biotium) and LSAB2 Streptavidin-HRP (K1016, Dako, Carpinteria, CA) according to the manufacturer's instructions. Apoptotic tubular cells were enumerated in 10 randomly chosen visual fields (magnification, ×400) of the sections, and the average of 10 selected microscopic fields was calculated. All measurements were recorded without a prior knowledge of the experimental procedures.

### Flow cytometry

Under the deep anesthesia, mice were perfused with 20 ml of ice-cold PBS to eliminate intravascular leukocytes, and the kidneys were obtained, minced into fragments of 1 mm^3^ and digested with 1 mg/ml of collagenase type 4 (Worthington Biochemical, Lakewood, NJ) and 100 U/ml DNase I (Worthington Biochemical) in opti-MEM medium for 45 min at 37 °C. After digestion, tissues pieces were then passed sequentially through a 100-μm and then a 40-μm filter. The cell suspension was centrifuged at 300 x *g*, for 5 min, and red blood cells were lysed using red blood cell lysis buffer (Imgenex, San Diego, CA). The single-cells were incubated with 25 μg/ml of Fc block (BD Biosciences Pharmingen, Piscataway, NJ) for 15 min at 4 °C. The cells were stained with eFluor 450-labeled rat anti-mouse Ly-6G Ab, FITC labeled rat anti-mouse CD3e, APC labeled rat anti-F4/80 Ab and Brilliant Violet 510-labeled rat anti-mouse CD45 Ab. The stained cells were analyzed on an CytoFLEX flow cytometer (Beckman Coulter Life Sciences, Indianapolis, IN), and the obtained data were analyzed using the FlowLogic (Inivai Technologies, Mentone, Australia).

### Isolation and culture of RPTECs

RPTECs were obtained from the BALB/c mice kidney as described previously^77^. Isolated RPTECs were cultured in renal epithelial growth media (REGM Bulletkit, Lonza Walkersville, Walkersville, MD) for 48 h, washed with PBS, and cultured in the same media for 4–5 days to reach 80% confluent and then treated with cisplatin and other reagents.

### Cell viability assay

The RPTECs were incubated in the presence of cisplatin (50 μmol/l) and TM (1 μg/ml) or PBS^78^. After 12 h culture, cell viability was assessed by colorimetric analysis of formazan (abs 450 nm) formation in viable cells using Cell Counting Kit-8 (DOJINDO Laboratories, Kumamoto, Japan).

### ROS detection in the RPTECs

The RPTECs were incubated in the presence of cisplatin (50 μmol/l) and TM (1 μg/ml) or PBS^78^. After 4 h culture, the cells were washed with HBSS and incubated with DCFH-DA (DOJINDO Laboratories) for 30 min according to the manufacturer’s instructions. Then the cells were fixed with 4% formaldehyde and permeated with 0.1% Triton x-100. After nuclear staining with DAPI, the cells were subjected to fluorescence microscopy analysis (KEYENCE BZ-X710, Osaka, Japan).

### Cellular ROS content detection by flow cytometry

Photo-oxidation Resistant DCFH-DA (FUJIFILM Wako Chemicals, Miyazaki, Japan) was used to measure superoxide anions in RPTECs. Confluent culture of RPTECs were treated cisplatin (50 μmol/l) with PBS or TM (1 μg/ml) for 4 h^78^. After washing cells three times with HBSS, RPTECs were incubated with Photo-oxidation Resistant DCFH-DA (100 pmol/l) for 30 min and trypsinized to obtain cell suspensions. After washing cells three times with PBS, cells were subjected to FACS analysis. DCFH-stained cells were analyzed using FlowLogic.

### Western blotting analysis of RPTECs or kidney samples

The RPTECs were cultured in the presence of cisplatin (50 μmol/l) with TM (1 μg/ml) or PBS^78^. Thereafter, Western blotting analyses of RPTECs were performed as described previously^34^. Briefly, the cells incubated with cold 10% trichloroacetic acid solution for 15 min on ice were scraped and centrifuged at 1500×*g* for 5 min. The cell pellets were dissolved with sodium dodecyl sulfate sample buffer. Equal amounts of protein were separated on 10 or 15% sodium dodecyl sulfate–polyacrylamide gel electrophoresis and transferred to an Immobilon-P transfer membrane (Millipore, Billerica, MA). The membranes were treated with primary Abs overnight at 4 °C and incubated for 1 h with a horseradish peroxidase-conjugated anti-rabbit secondary Ab (1:2000 dilution; Dako, Carpinteria, CA) at room temperature for 1 h. Bound Ab complexes were detected using an enhanced chemiluminescence reagent (Millipore) according to the manufacturer’s instructions. In another series, kidney samples were subjected to Western blotting analyses as described previously^34^. Briefly, tissue samples were homogenized with a lysis buffer (10 mmol/l PBS, pH 7.4 containing 0.01% Triton X-100, 0.5% sodium deoxycholate and 0.1% sodium dodecyl sulfate) containing Complete Protease Inhibitor Mixture, and Phosphatase Inhibitor Cocktails for serine/threonine protein phosphatases and tyrosine protein phosphatases (P2850 and P5726; Sigma-Aldrich) and centrifuged to obtain lysates. The lysates were analyzed as described above. The band intensities were measured using NIH Image Analysis Software version 1.61 (National Institutes of Health).

### Enzyme-linked immunosorbent assay (ELISA)

Kidney samples were homogenized with extraction buffer and centrifuged at 5000×*g* for 10 min, and the supernatant was subjected to ELISA. IFN-γ, Heme oxygenase (HO-1) levels were measured with a commercially available ELISA Kit (Abcam) according to the manufacturer's instructions. The detection limits for each method were as follows: IFN‐γ > 0.04 pg/ml; HO-1 > 0.04 pg/ml. Total protein in each supernatant was measured with a commercially available kit (BCA Protein Assay Kit; Pierce, MO). Data were expressed as a value per protein (pg/mg) for each sample.

### Extraction of total RNA and real-time RT-PCR

Real-time RT-PCR was performed as described previously^79,80^. Briefly, total RNA was extracted from whole kidney samples using ISOGENE (Nippon Gene, Toyama, Japan) according to the manufacturer’s instructions, and 1 μg of total RNA was reverse-transcribed into cDNA at 42 °C for 1 h in 20 μl of a reaction mixture containing mouse Moloney leukemia virus reverse transcriptase (PrimeScript, Takara Bio Inc., Otsu, Japan) with 6 random primers (Takara Bio Inc.). The generated cDNA was subjected to a real-time PCR analysis using SYBR^®^ Premix Ex Taq™ II kit (Takara Bio Inc.) with specific primer sets (Table 1). The relative quantity of target gene expression to *Actb* was measured by a comparative Ct method.

**Table 1:** Sequences of primers used for real-time RT-PCR.

Transcript	Sequence
*Ifng*	(F) 5′-CGGCACAGTCATTGAAAGCCTA -3′ (R) 5′-GTTGCTGATGGCCTGATTGTC-3′
*Hmox*	(F) 5′-TGCAGGTGATGCTGACAGAGG-3′ (R) 5′-GGGATGAGCTAGTGCTGATCTGG-3′
*Tnfa*	(F) 5′-AAGCCTGTAGCCCACGTCGTA -3′ (R) 5′-GGCACCACTAGTTGGTTGTCTTTG-3′
*Il1b*	(F) 5′-TCCAGGATGAGGACATGAGCAC -3′ (R) 5′-GAACGTCACACACCAGCAGGTTA-3′
*Cxcl1*	(F) 5′-TGCACCCAAACCGAAGTC -3′ (R) 5′-GTCAGAAGCCAGCGTTCACC-3′
*Cxcl2*	(F) 5′-AAAGTTTGCCTTGACCCTGAA-3′ (R) 5′-CTCAGACAGCGAGGCACATC-3′
*Actb*	(F) 5′-CATCCGTAAAGACCTCTATGCCAAC-3′ (R) 5′-ATGGAGCCACCGATCCACA-3′

(F), Forward primer; (R), Reverse primer.

### Statistical analysis

Data were expressed as the mean ± SEM. For the comparison between control group and TM group at multiple time points, 2-way ANOVA followed by Dunnett’s post-hoc test was used. To compare the values between two groups, a two-sided unpaired Student’s *t* test was performed. In the series of cisplatin treatment on RPTECs in vitro, one-way ANOVA followed by Dunnett’s post hoc test was used. *P* < 0.05 was considered statistically significant. All statistical analyses were performed using Statcel3 software.

### Ethics

All animal experiments were approved by the Committee on Animal Care and Use at Wakayama Medical University (approval number 1095), and all methods were performed in accordance with relevant regulations and guidelines including the ARRIVE guideline.

### Supplementary Information

Supplementary Figures.

## Data Availability

The authors declare that all data are available in the article file or available from the corresponding authors, Yuko Ishida and Toshikazu Kondo, upon request.
