# Supplementary material for: Protective roles of thrombomodulin in cisplatin-induced nephrotoxicity through the inhibition of oxidative and endoplasmic reticulum stress
Source: Sci Rep. 2024 Jun 18;14:14004. doi: 10.1038/s41598-024-64619-y (PMC11189513; doi:10.1038/s41598-024-64619-y)
Supplement: Supplementary file 1 — Supplementary Figures. [file 41598_2024_64619_MOESM1_ESM.docx]

**Supplementary Information**

**Protective roles of thrombomodulin in cisplatin-induced nephrotoxicity through the inhibition of oxidative and endoplasmic reticulum stress**

Hiroki Yamamoto^1,†^, Yuko Ishida^1,†,^*, Siying Zhang^1^, Miyu Osako^1^, Mizuho Nosaka^1^, Yumi Kuninaka^1^, Akiko Ishigami^1^, Yuya Iwahashi^1,2^, Miki Aragane^1^, Lennon Matsumoto^1^, Akihiko Kimura^1^, Toshikazu Kondo^1,^*

^1^Department of Forensic Medicine, Wakayama Medical University, Wakayama, Japan

^2^Department of Urology, Wakayama Medical University, Wakayama, Japan

**^†^**Both authors equally contributed to this work.

***Corresponding:** Dr. Yuko Ishida, PhD and Dr. Toshikazu Kondo, MD & PhD

Department of Forensic Medicine, Wakayama Medical University,

811-1 Kimiidera, Wakayama 641-0012, Japan

E-mail: iyuko@wakayama-med.ac.jp and kondot@wakayama-med.ac.jp

Phone & Fax: +81-73-441-0641


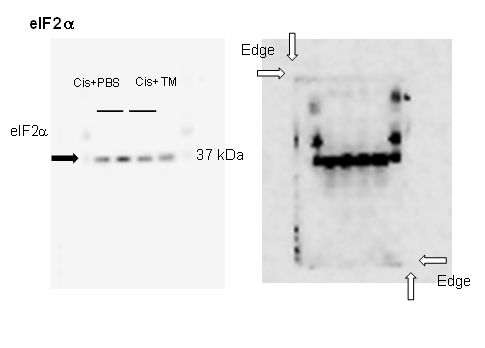

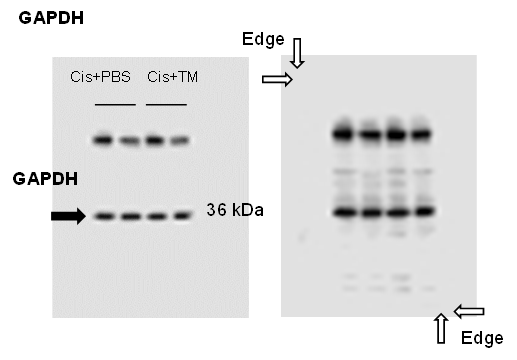


**Supplementary Fig. S1.**The uncropped original images in Fig. 6c.

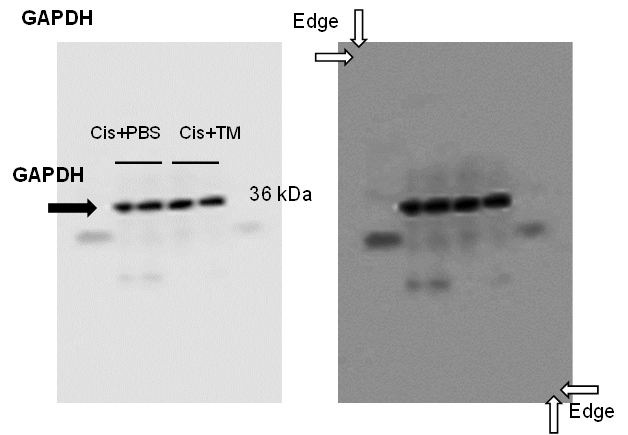


**Supplementary Fig. S2.**The uncropped original images in Fig. 7a.

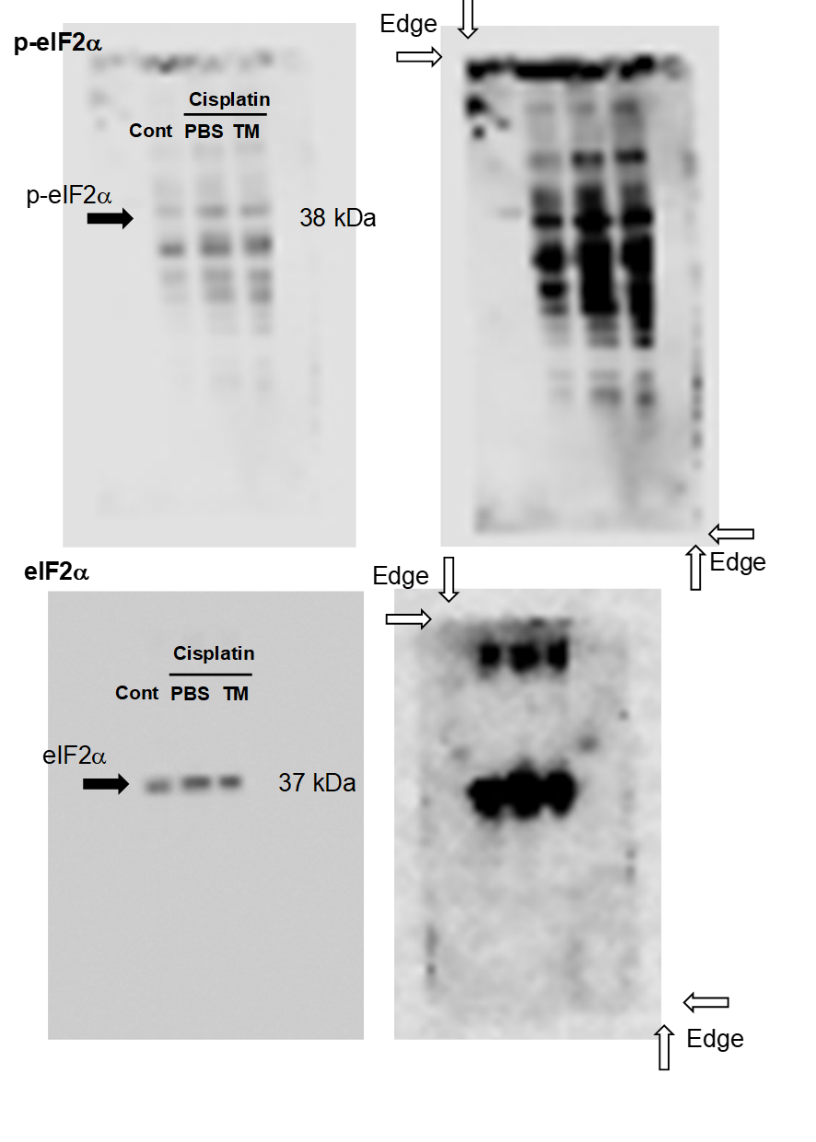

**Supplementary Fig. S3.**The uncropped original images in Fig. 8b.
